# Targeting the IL-5 pathway in eosinophilic asthma: a comparison of mepolizumab to benralizumab in the reduction of peripheral eosinophil counts

**DOI:** 10.1186/s13223-020-00507-0

**Published:** 2021-01-06

**Authors:** Arian Ghassemian, Jane Jiyoon Park, Michael W. Tsoulis, Harold Kim

**Affiliations:** 1 Division of Clinical Immunology and Allergy, Department of Medicine, Schulich School of Medicine and Dentistry, Western University, London, ON Canada; 2 Renaissance School of Medicine, Stony Brook University, Stony Brook, NY USA; 3 Division of Clinical Immunology and Allergy, Department of Medicine, Michael D.DeGroot School of Medicine, McMaster University, Hamilton, ON Canada

**Keywords:** Asthma, Biologics, Interlukin-5, Interlukin-5 receptor, Benralizumab, Mepolizumab

## Abstract

**Background:**

Mepolizumab and benralizumab are biologics approved for severe eosinophilic asthma. Mepolizumab is an anti-interlukin-5 (IL-5) antibody while benralizumab is an anti-interleukin-5 receptor alpha (IL-5Rα) antibody targeting the IL-5 receptor on eosinophils. Both therapies reduce oral corticosteroid requirements and asthma exacerbations. However, no head-to-head studies have been published. The aim of the present study was to compare the efficacy of peripheral eosinophil reduction of mepolizumab and benralizumab.

**Methods:**

A retrospective chart review was conducted on patients with severe eosinophilic asthma who were approved for either IL-5 agent. Patients with noted non-adherence or those who were on fluctuating doses of corticosteroids for non-asthma related illnesses were excluded. The last detectable eosinophil count for each patient prior to start of therapy was compared to the highest eosinophil count noted after therapy start with at least 30 days of adherence.

**Results:**

Thirty-six patients taking mepolizumab and 19 patients taking benralizumab met the inclusion criteria and had both pre-treatment and post-treatment eosinophil counts. Baseline characteristics were not statistically different between those on mepolizumab and benralizumab therapy. The mean pre-therapy serum eosinophil count did not statistically differ between patients on mepolizumab (597.2 cells/µL) compared to benralizumab (521.6 cells/µL), p = 0.3769. While both therapies resulted in a significant decrease in eosinophil count (*p* < 0.0001); the mean decrease did not statistically differ between patients taking mepolizumab compared to those on benralizumab, p = 0.9079. Nonetheless, 100% of patients receiving benralizumab had undetectable eosinophil counts post-therapy compared to 31% of patients receiving mepolizumab (*p* < 0.0001).

**Conclusion:**

Both mepolizumab and benralizumab are potent targets of the IL-5 pathway with the ability to significantly reduce peripheral eosinophil counts. While there is there is no statistical difference in the magnitude of eosinophil reduction offered by each agent, benralizumab is able to decrease peripheral eosinophil counts to 0 cells/µL in more patients than mepolizumab.

## Background

Over the past decade, the heterogeneity of asthma has become better appreciated and characterized [1]. Currently, there are two broad categories of asthma endotypes, namely type 2 and non-type 2. Type 2, also known as eosinophilic asthma, is a helper T cell 2 (Th2) mediated process, the hallmark of which is high blood and sputum eosinophil count [2, 3]. In the recent past, treatment options for patients with severe persistent type 2 asthma refractory to medical therapy were limited. However, with the advent of biologic therapies, this patient population currently has four targeted monoclonal antibodies approved by Health Canada. Specifically, mepolizumab and reslizumab target interleukin 5 (IL-5) while benralizumab targets the IL-5 receptor alpha (IL-5Rα) subunit.

IL-5 is a Th2 cytokine that is involved in eosinophil activation, maturation and survival [4, 5]. All IL-5 therapies have been shown to reduce oral corticosteroid use and overall asthma exacerbations [6], but there is speculation as to whether benralizumab may have clinical superiority due to its unique ability to deplete eosinophils compared to direct IL-5 antibodies [7]. Although there have been multiple indirect systematic reviews and meta-analyses comparing the effects of various biologics, the data are inconsistent [7–9]. Thus far, there have been no head-to-head comparisons of these biologic agents with respect to peripheral eosinophil reduction. Both mepolizumab and benralizumab are subcutaneously administered monoclonal antibodies that target the IL-5 pathway, albeit through different mechanisms of action. It is unclear if this has a differential effect on peripheral eosinophil reduction. Therefore, the aim of the present study was to determine if the peripheral eosinophil reduction efficacy of mepolizumab and benralizumab differ.

## Methods

A retrospective chart review was conducted on patients with severe eosinophilic asthma at a specialist referral clinic in Kitchener, Ontario who were approved for either mepolizumab or benralizumab. All patients on mepolizumab and benralizumab therapy were reviewed for consideration of inclusion in the study. Patients with noted non-adherence or those who were on fluctuating doses of corticosteroids for non-asthma related illnesses were excluded. For all patients, the pre-treatment eosinophil counts were reviewed and the last detectable eosinophil count prior to the therapy start date (or where unavailable, the eosinophil count used for drug approval) was recorded as the patient’s baseline pre-eosinophil count. The post-treatment eosinophil count was the highest eosinophil count recorded after therapy start date with at least 30 days of adherence during data collection period as all available values were reviewed. In the case of multiple equivalent eosinophil counts post-treatment initiation, the eosinophil count closest to therapy onset with at least 30 days of adherence was utilized to determine time between start of therapy and post-therapy eosinophil counts. Once data collection was complete, the pre-treatment eosinophil and post-treatment eosinophil counts were compared.

A total of 5 participants switched from the mepolizumab arm to the benralizumab arm. No participants switched from benralizumab to mepolizumab. One of these patients lacked a post-therapy eosinophil count after beginning benralizumab therapy and was therefore excluded from the benralizumab arm. The reason for these participants switching was not based on rising eosinophil counts but due to inadequate control of asthma or intolerance to the therapy. A comparison of the patients on mepolizumab who switched to benralizumab versus patients on mepolizumab who did not switch therapies revealed no significant differences (Additional file 1: Table S1). Therefore, those patients who switched were included in the final mepolizumab group. However, compared to patients on benralizumab who did not change therapies, patients on benralizumab who switched from mepolizumab had significantly lower pre-eosinophil counts (Additional file 2: Table S2). These patients were included in the final benralizumab group as the analysis without these patients yielded similar results (Additional file 3: Table S3) as those in the final analysis.

For patients in the mepolizumab arm who switched to benralizumab, the pre-therapy eosinophil count was collected as described above. The post-therapy eosinophil count was the highest detectable counts prior to start of benralizumab.

For patients in the benralizumab arm who switched from mepolizumab, the pre-therapy eosinophil value was the last detectable eosinophil count prior to start of benralizumab. The post-therapy eosinophil value was the highest eosinophil count recorded after therapy start date during data collection as all available values were reviewed.

This study protocol was approved by the Hamilton Integrated Research Ethics Board --5437-C.

### Data analysis

A Student t-test or Mann–Whitney test, where appropriate based on normality, was used to compare the baseline characteristic means of patients on mepolizumab and benralizumab. Wilcoxon signed ranked test was used to compared pre- and post-therapy eosinophil counts for patients treated with mepolizumab and benralizumab. Chi-Square Test for Independence or Fisher’s Exact Test for Independence, where appropriate, was used to analyze the association between categorical variables. The Kaplan–Meier method was utilized to assess the percentage of patients with eosinophil counts greater than zero over time between mepolizumab and benralizumab (event defined as an eosinophil count of zero). p values < 0.05 were considered statistically significant.

## Results

**Fig. 1:**
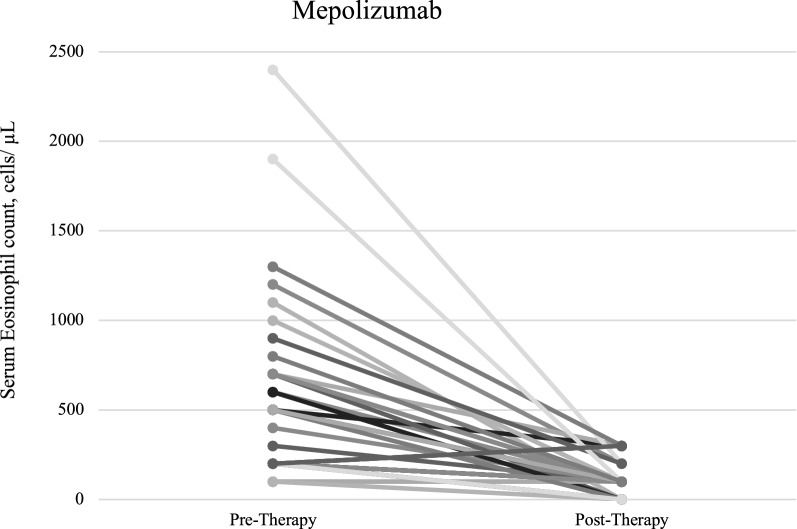
Mepolizumab pre-therapy and post-therapy serum eosinophil count, cells/μL

**Fig. 2:**
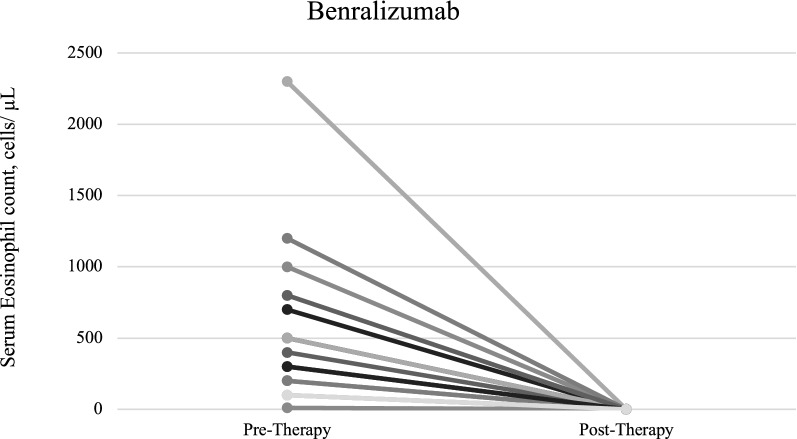
Benralizumab pre-therapy and post-therapy serum eosinophil count, cells/μL

Thirty-six patients taking mepolizumab and 19 patients taking benralizumab met the inclusion criteria and had both pre-treatment and post-treatment eosinophil counts. There was no statistically significant difference between the age, sex, smoking history, mean number of comorbidities, anaphylaxis history, non-asthma atopic disease, food or environmental allergies, family history of atopic disease, mean age of asthma onset, and mean number of therapies prior to start of biologic therapy of patients taking mepolizumab versus benralizumab (Table 1). The mean pre-therapy serum eosinophil count did not statistically differ between patients on mepolizumab (597.2 cells/µL) compared to benralizumab (521.6 cells/µL), p = 0.376. The percentage of patients with eosinophilia (≥ 500 cells/µL) prior to therapy onset was similar between groups, p = 0.2730. While both therapies resulted in a significant decrease in eosinophil count (*p* < 0.0001), the mean decrease did not statistically differ between patients taking mepolizumab compared to those on benralizumab, p = 0.9079 (Table 2, Figs. 1, 2). Furthermore, all patients with eosinophilia prior to therapy onset had post-eosinophil counts within normal limits (< 500 cells/µL). Nonetheless, 100% of patients receiving benralizumab had undetectable eosinophil counts post-therapy compared to 31% of patients receiving mepolizumab (p < 0.0001) (Table 2). Moreover, two patients in the mepolizumab group had elevations in their serum eosinophil count post-therapy. Specifically, these two patients’ serum eosinophil counts went from 200 cells/µL to 300 cells/µL (Fig. 1).

**Table 1:** Baseline characteristics between treatment groups

Characteristic	Mepolizumab (n = 36)	Benralizumab (n = 19)	p-value
Mean age (range)	53.8 (33–79)	59.6 (23–77)	0.0839
Sex			0.7028
Female, n (%)	17 (47)	10 (53)	
Male, n (%)	19 (53)	9 (47)	
Smoking history			0.8406
Never, n (%)	23 (64)	11 (58)	
Former, n (%)	7 (19)	5 (26)	
Active, n (%)	3 (8)	3 (16)	
No history available, n (%)	3 (8)	0 (0)	
Mean # of comorbidities	2.3 (0–9)	3.2 (0–9)	0.1363
Comorbid lung disease			0.7652
Yes, n (%)	10 (28)	6 (32)	
No, n (%)	26 (72)	13 (68)	
Anaphylaxis history			0.0753
Yes, n (%)	4 (11)	6 (32)	
No, n (%)	28 (78)	13 (68)	
No history available, n (%)	4 (11)	0 (0)	
Non-asthma atopic disease Yes, n (%)	25 (69)	14 (74)	0.6208
No, n (%)	2 (6)	2 (11)	
No history available, n (%)	9 (25)	3 (16)	
Food/environmental allergy			0.4649
Yes, n (%)	21 (58)	11 (58)	
No, n (%)	5 (14)	5 (26)	
No history available, n (%)	10 (28)	2 (11)	
Family history of atopic disease			0.2594
Yes, n (%)	15 (42)	12 (63)	
No, n (%)	18 (50)	7 (37)	
No history available, n (%)	3 (8)	0 (0)	
Mean age of asthma onset (range)	36.4 (13–59)	45.8 (13–72)	0.1800
No history available, n (%)	21 (58)	8 (42)	
Mean # of therapies prior to biologic (range)	3.8 (2–8)	3.5 (1–5)	0.6875

**Table 2:** Peripheral eosinophil reduction post therapy initiation

Measure	Mepolizumab (n = 36)	Benralizumab (n = 19)	p-value
Pre-therapy serum eosinophil count, cells/µL, mean (SD)	597.2 (504.5)	521.6 (546.8)	0.3769
Patients with pre-therapy eosinophilia (≥ 500 cells/µL)			0.2730
Yes, n (%)	21 (58)	8 (42)	
No, n (%)	15 (42)	11 (58)	
Post-therapy serum eosinophil count, cells/µL, mean (SD)	103.1 (100.0)	0 (0)	−
Decrease in serum eosinophil count, cells/µL, mean (SD)	494.1 (492.9)	521.6 (546.8)	0.9079
Patients with undetectable eosinophil count post-therapy			< 0.0001
Yes, n (%)	11 (31)	19 (100)	
No, n (%)	25 (69)	0 (0)	
Patients with pre-therapy eosinophilia (≥ 500 cells/µL) who have normal eosinophil counts (< 500 cells/µL) post-therapy			-
Yes, n (%)	21 (100)	8 (100)	
No, n (%)	0 (0)	0 (0)	
Time from therapy onset to post-therapy serum eosinophil count, days, mean (SD)	280.4 (191.9)	118.7 (62.2)	0.0066

**Fig. 3:**
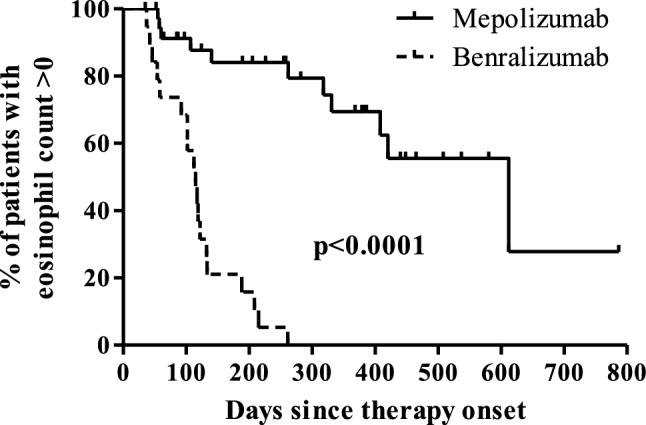
Kaplan–Meier product limit estimates of percentages of patients with eosinophil counts greater than zero over time on mepolizumab and menralizumab therapies

Time from therapy onset to post-eosinophil count was longer in the mepolizumab group compared to the benralizumab group (p = 0.0066) (Table 2). A log rank test was conducted to determine if there was a difference in Kaplan–Meier product limit estimates of percentages of patients with eosinophil counts greater than zero over time between mepolizumab and benralizumab (Fig. 3). The distributions for the two therapies were statistically different, χ^2^(1) = 37.91, p < 0.0001.

Comorbid lung disease includes Chronic Obstructive Pulmonary Disease (COPD), Eosinophilic Pneumonia, Obstructive Sleep Apnea (OSA), Allergic Bronchopulmonary Aspergillosis (ABPA), Bronchiectasis, Eosinophilic Granulomatosis with Polyangiitis (EGPA). Non-Asthma atopic disease includes Allergic Rhinitis, Nasal Polyposis and Conjunctivitis. Food/environmental allergy includes positive skin testing and/or positive history of symptoms with exposure.

## Discussion

IL-5 has been a focus of treatment research for severe eosinophilic asthma for the past decade due to its role in eosinophil activation, maturation and survival [6]. Previous studies have shown that both benralizumab and mepolizumab have been successful at reducing number of asthma exacerbations as well as peripheral eosinophil counts [7–9]. While mepolizumab acts on IL-5 cytokines and inhibits the activation and differentiation of eosinophils [10, 11], benralizumab acts by binding to the IL-5Rα subunit on eosinophils. This not only prevents signal transduction but also triggers cell-mediated killing by the innate immune system resulting in eosinophil depletion [12, 13]. Several indirect meta-analyses comparing the treatment groups have been performed but with conflicting data [7–9]. While Menzella et al. found that benralizumab may be superior to mepolizumab in reducing oral corticosteroid use [7], Busse et al. found that mepolizumab was associated with greater improvement in exacerbations and asthma control compared with reslizumab or benralizumab [8]. In contrast, Cabon et al. reported that there was no clear superiority between the drugs when appropriate doses were compared [9]. Current Global Initiative for Asthma clinical guidelines released in 2019 do not provide guidance on selecting the various IL5 treatment options [14]. The present study aimed to compare these two biologics commonly used to treat severe Type 2 asthma to better elucidate meaningful differences between the therapies.

The present study shows that although there was no statistical difference in the magnitude of reduction in peripheral eosinophil counts between benralizumab and mepolizumab, benralizumab was able to decrease eosinophil counts to zero in 100% of cases compared to 31% of mepolizumab cases. This is consistent with previous studies that have shown the ability of benralizumab to decrease peripheral eosinophil counts to zero [15, 16]. Furthermore, patients on benralizumab appear to reach an eosinophil count of zero significantly quicker than patients on mepolizumab, although this measure is limited by unstandardized follow-up. These differential effects are likely related to the distinct mechanism of action by which benralizumab affects the IL-5Rα subunit on eosinophils. Moreover, in patients with eosinophilia (≥ 500 cells/µL) prior to therapy onset, both mepolizumab and benralizumab were able to decrease the eosinophil count to within normal limits (< 500 cells/µL) post-therapy in all cases.

The clinical implications of the magnitude of eosinophil, reduction to zero, reduction within normal limits and time to reduction to zero remain unknown. Although it has been established that eosinophils play a vital role in asthma symptoms and control, it is not yet fully understood if there is a linear correlation between peripheral eosinophil counts and control of eosinophilic asthma. Moreover, it is unclear the clinical implications of the two patients in the mepolizumab group that had elevations in their peripheral eosinophil count post-therapy.

Limitations of this retrospective study include low sample size and selection bias as patients were not randomly allocated to receive mepolizumab or benralizumab. Although there were no statistical differences between the two groups when assessing age, sex, smoking history, mean number of comorbidities, anaphylaxis history, non-asthma atopic disease, food or environmental allergies, family history of atopic disease, mean age of asthma onset, and mean number of therapies prior to start of biologic therapy, there were patients without histories available which limited these comparisons. Moreover, it is possible that the two groups differed with regards to important confounders that were not able to be measured due to the retrospective nature of the study. Additionally, correlation of eosinophil depletion with clinical outcomes, such as measures of asthma control, effect on asthma exacerbation rates, changes in FEV1, or other biomarkers of asthma control—such as sputum eosinophil count or fractional exhaled nitric oxide was not possible as the timing of the measurement of these clinical outcomes post-therapy onset was highly variable between patients, if these measurements were even available. Lastly, the time between therapy onset and post-therapy eosinophil count may not be indicative of the time each agent acts to decrease eosinophil count as this time could be confounded my patient delay or lack of follow-up for bloodwork.

## Conclusion

The present study demonstrates that benralizumab is more potent in its suppression of peripheral eosinophils, with counts of 0 cells/μL in all cases post therapy initiation. Future studies evaluating the clinical significance of this noted difference between these therapies are warranted.

## Supplementary Information

**Additional file 1: Table S1.** Comparison of patients who switched from mepolizumab to benralizumab versus patients on mepolizumab who did not switch.

**Additional file 2: Table S2.** Comparison of patients who switched from mepolizumab to benralizumab versus patients on benralizumab who did not switch.

**Additional file 3: Table S3.** Comparison of patients on mepolizumab versus patients on benralizumab who did not switch from mepolizumab.

## Data Availability

The datasets used and/or analysed during the current study are available from the corresponding author on reasonable request.

## Abbreviations

Th2: Helper T cell 2
IL-5: Interleukin 5
IL-5Rα: Interleukin-5 receptor alpha
