# Supplementary material for: Targeting the IL-5 pathway in eosinophilic asthma: a comparison of mepolizumab to benralizumab in the reduction of peripheral eosinophil counts
Source: Allergy Asthma Clin Immunol. 2021 Jan 6;17:3. doi: 10.1186/s13223-020-00507-0 (PMC7789431; doi:10.1186/s13223-020-00507-0)
Supplement: Supplementary file 1 — Additional file 1: Table S1. Comparison of patients who switched from mepolizumab to benralizumab versus patients on mepolizumab who did not switch. [file 13223_2020_507_MOESM1_ESM.docx]

**Additional file 1: Table S1.** Comparison of patients who switched from mepolizumab to benralizumab versus patients on mepolizumab who did not switch

| Characteristic | Mepolizumab – patients who switched  (n=5) | Mepolizumab – patients who did not switch  (n=31) | p-value |
| --- | --- | --- | --- |
| Mean age (range) | 59.0 (44-65) | 52.9 (33-79) | 0.2513 |
| Sex |  |  | 0.6504 |
| Female, n (%) | 3 (60) | 14 (45) |  |
| Male, n (%) | 2 (40) | 17 (55) |  |
| Smoking history |  |  | 0.6521 |
| Never, n (%)  Former, n (%)  Active, n (%) | 3 (60)  1 (20)  1 (20) | 20 (65)  6 (19)  2 (6) |  |
| No history available, n (%) | 0 (0) | 3 (10) |  |
| Mean # of comorbidities | 4.2 (0-9) | 1.97 (0-9) | 0.3860 |
| Comorbid lung disease |  |  | 1.0000 |
| Yes, n (%)  No, n (%) | 1 (20)  4 (80) | 9 (29)  22 (71) |  |
| Anaphylaxis history |  |  | 0.1053 |
| Yes, n (%)  No, n (%)  No history available, n (%) | 2 (40)  3 (60)  0 (0) | 2 (6)  25 (81)  4 (13) |  |
| Non-asthma atopic disease  Yes, n (%)  No, n (%)  No history available, n (%) | 3 (60)  0 (0)  2 (40) | 22 (71)  2 (6)  7 (23) | 1.0000 |
| Food/environmental allergy |  |  | 0.4885 |
| Yes, n (%)  No, n (%)  No history available, n (%) | 2 (40)  1 (20)  2 (40) | 19 (61)  4 (13)  8 (26) |  |
| Family history of atopic disease  Yes, n (%)  No, n (%)  No history available, n (%) | 2 (40)  2 (40)  1 (20) | 13 (42)  16 (52)  2 (6) | 1.0000 |
| Mean age of asthma onset (range) | 32.0 (16-52) | 38.0 (13-59) | 0.4330 |
| No history available, n (%) | 1 (20) | 20 (65) |  |
| Mean # of therapies prior to biologic (range) | 4.0 (3-5) | 3.8 (2-8) | 0.5895 |
| Pre-therapy serum eosinophil count, cells/µL, mean (SD) | 440.0 (251.0) | 622.6 (532.7) | 0.6425 |
| Post-therapy serum eosinophil count, cells/µL, mean (SD) | 82.0 (128.9) | 106.5 (96.4) | 0.5107 |
| Patients with pre-therapy eosinophilia (≥ 500 cells/µL)  Yes, n (%)  No, n (%) | 3 (60)  2 (40) | 18 (58)  13 (42) | 1.0000 |
| Decrease in serum eosinophil count, cells/µL, mean (SD) | 358.0 (332.9) | 516.1 (514.5) | 0.6802 |
| Patients with undetectable eosinophil count post-therapy  Yes, n (%)  No, n (%) | 2 (40)  3 (60) | 9 (29)  22 (71) | 0.6309 |
| Patients with pre-therapy eosinophilia (≥ 500 cells/µL) and normal count (<500 cells/µL) post-therapy  Yes, n (%)  No, n (%) | 3 (100)  0 (0) | 18 (100)  0 (0) | - |
| Time from therapy onset to post-therapy serum eosinophil count, days, mean (SD) | 209.2 (196.9) | 291.9 (191.9) | 0.3484 |
