# Supplementary material for: Targeting the IL-5 pathway in eosinophilic asthma: a comparison of mepolizumab to benralizumab in the reduction of peripheral eosinophil counts
Source: Allergy Asthma Clin Immunol. 2021 Jan 6;17:3. doi: 10.1186/s13223-020-00507-0 (PMC7789431; doi:10.1186/s13223-020-00507-0)
Supplement: Supplementary file 2 — Additional file 2: Table S2. Comparison of patients who switched from mepolizumab to benralizumab versus patients on benralizumab who did not switch. [file 13223_2020_507_MOESM2_ESM.docx]

**Additional file 2: Table S 2.** Comparison of patients who switched from mepolizumab to benralizumab versus patients on benralizumab who did not switch

| Characteristic | Benralizumab – patients who switched  (n=4) | Benralizumab – patients who did not switch  (n=15) | p-value |
| --- | --- | --- | --- |
| Mean age (range) | 62.8 (58-65) | 58.80 (23-77) | 0.6123 |
| Sex |  |  | 1.0000 |
| Female, n (%) | 2 (50) | 8 (53) |  |
| Male, n (%) | 2 (50) | 7 (47) |  |
| Smoking history |  |  | 0.5888 |
| Never, n (%)  Former, n (%)  Active, n (%) | 3 (75)  1 (25)  0 (0) | 8 (53)  4 (27)  3 (20) |  |
| No history available, n (%) | 0 (0) | 0 (0) |  |
| Mean # of comorbidities | 4.5 (0-9) | 2.9 (0-9) | 0.3879 |
| Comorbid lung disease |  |  | 1.0000 |
| Yes, n (%)  No, n (%) | 1 (25)  3 (75) | 5 (33)  10 (67) |  |
| Anaphylaxis history |  |  | 0.5573 |
| Yes, n (%)  No, n (%)  No history available, n (%) | 2 (50)  2 (50)  0 (0) | 4 (27)  11 (73)  0 (0) |  |
| Non-asthma atopic disease  Yes, n (%)  No, n (%)  No history available, n (%) | 2 (50)  0 (0)  2 (50) | 12 (80)  2 (13)  1 (7) | 1.0000 |
| Food/environmental allergy |  |  | 0.2143 |
| Yes, n (%)  No, n (%)  No history available, n (%) | 1 (25)  2 (50)  1 (25) | 10 (67)  3 (20)  1 (7) |  |
| Family history of atopic disease  Yes, n (%)  No, n (%)  No history available, n (%) | 2 (50)  2 (50)  0 (0) | 10 (67)  5 (23)  0 (0) | 0.6027 |
| Mean age of asthma onset (range) | 33.5 (16-52) | 52.9 (13-72) | 0.1636 |
| No history available, n (%) | 0 (0) | 8 (53) |  |
| Mean # of therapies prior to biologic (range) | 3.5 (3-5) | 3.5 (1-5) | 0.7530 |
| Pre-therapy serum eosinophil count, cells/µL, mean (SD) | 127.5 (122.6) | 626.7 (570.0) | 0.0236 |
| Patients with pre-therapy eosinophilia (≥ 500 cells/µL)  Yes, n (%)  No, n (%) | 0 (0)  4 (100) | 8 (53)  7 (47) | 0.1032 |
| Post-therapy serum eosinophil count, cells/µL, mean (SD) | 0 (0) | 0 (0) | - |
| Decrease in serum eosinophil count, cells/µL, mean (SD) | 127.5 (122.6) | 626.7 (570.0) | 0.0236 |
| Patients with undetectable eosinophil count post-therapy  Yes, n (%)  No, n (%) | 4 (100)  0 (0) | 15 (100)  0 (0) | - |
| Patients with pre-therapy eosinophilia (≥ 500 cells/µL) and normal counts (<500 cells/µL) post-therapy  Yes, n (%)  No, n (%) | -  - | 8 (100)  0 (0) | - |
| Time from therapy onset to post-therapy serum eosinophil count, days, mean (SD) | 112.0 (18.3) | 120.5 (70.0) | 1.0000 |
