# Supplementary material for: Targeting the IL-5 pathway in eosinophilic asthma: a comparison of mepolizumab to benralizumab in the reduction of peripheral eosinophil counts
Source: Allergy Asthma Clin Immunol. 2021 Jan 6;17:3. doi: 10.1186/s13223-020-00507-0 (PMC7789431; doi:10.1186/s13223-020-00507-0)
Supplement: Supplementary file 3 — Additional file 3: Table S3. Comparison of patients on mepolizumab versus patients on benralizumab who did not switch from mepolizumab. [file 13223_2020_507_MOESM3_ESM.docx]

**Additional file 3: Table S3.** Comparison of patients on mepolizumab versus patients on benralizumab who did not switch from mepolizumab

| Characteristic | Mepolizumab  (n=36) | Benralizumab – patients who did not switch  (n=15) | p-value |
| --- | --- | --- | --- |
| Mean age (range) | 53.8 (33-79) | 58.80 (23-77) | 0.1834 |
| Sex |  |  | 0.6908 |
| Female, n (%) | 17 (47) | 8 (53) |  |
| Male, n (%) | 19 (53) | 7 (47) |  |
| Smoking history |  |  | 0.4623 |
| Never, n (%)  Former, n (%)  Active, n (%) | 23 (64)  7 (64)  3 (8) | 8 (53)  4 (27)  3 (20) |  |
| No history available, n (%) | 3 (8) | 0 (0) |  |
| Mean # of comorbidities | 2.3 (0-9) | 2.9 (0-9) | 0.2500 |
| Comorbid lung disease |  |  | 0.7428 |
| Yes, n (%)  No, n (%) | 10 (28)  26 (72) | 5 (33)  10 (67) |  |
| Anaphylaxis history |  |  | 0.2455 |
| Yes, n (%)  No, n (%)  No history available, n (%) | 4 (11)  28 (78)  4 (11) | 4 (27)  11 (73)  0 (0) |  |
| Non-asthma atopic disease  Yes, n (%)  No, n (%)  No history available, n (%) | 25 (69)  2 (6)  9 (25) | 12 (80)  2 (13)  1 (7) | 0.5956 |
| Food/environmental allergy |  |  | 1.0000 |
| Yes, n (%)  No, n (%)  No history available, n (%) | 21 (58)  5 (14)  10 (28) | 10 (67)  3 (20)  1 (7) |  |
| Family history of atopic disease  Yes, n (%)  No, n (%)  No history available, n (%) | 15 (42)  18 (50)  3 (8) | 10 (67)  5 (23)  0 (0) | 0.2209 |
| Mean age of asthma onset (range) | 36.4 (13-59) | 52.9 (13-72) | 0.0482 |
| No history available, n (%) | 21 (58) | 8 (53) |  |
| Mean # of therapies prior to biologic (range) | 3.8 (2-8) | 3.5 (1-5) | 0.7901 |
| Pre-therapy serum eosinophil count, cells/µL, mean (SD) | 597.2 (504.5) | 626.7 (570.0) | 0.9169 |
| Patients with pre-therapy eosinophilia (≥ 500 cells/µL)  Yes, n (%)  No, n (%) | 21 (58)  15 (42) | 8 (53)  7 (47) | 0.7657 |
| Post-therapy serum eosinophil count, cells/µL, mean (SD) | 103.1 (100.0) | 0 (0) | - |
| Decrease in serum eosinophil count, cells/µL, mean (SD) | 494.2 (492.6) | 626.7 (570.0) | 0.9079 |
| Patients with undetectable eosinophil count post-therapy  Yes, n (%)  No, n (%) | 11 (31)  25 (69) | 15 (100)  0 (0) | <0.0001 |
| Patients with pre-therapy eosinophilia (≥ 500 cells/µL) and normal counts (<500 cells/µL) post-therapy  Yes, n (%)  No, n (%) | 21 (100)  0 (0) | 8 (100)  0 (0) | - |
| Time from therapy onset to post-therapy serum eosinophil count, days, mean (SD) | 280.4 (191.9) | 120.5 (70.0) | 0.0058 |
